# Regulation of Axolotl (*Ambystoma mexicanum*) Limb Blastema Cell Proliferation by Nerves and BMP2 in Organotypic Slice Culture

**DOI:** 10.1371/journal.pone.0123186

**Published:** 2015-04-29

**Authors:** Jeffrey Lehrberg, David M. Gardiner

**Affiliations:** Department of Developmental and Cell Biology, University of California Irvine, Irvine, California, United States of America; National Cancer Institute, UNITED STATES

## Abstract

We have modified and optimized the technique of organotypic slice culture in order to study the mechanisms regulating growth and pattern formation in regenerating axolotl limb blastemas. Blastema cells maintain many of the behaviors that are characteristic of blastemas *in vivo* when cultured as slices *in vitro*, including rates of proliferation that are comparable to what has been reported *in vivo*. Because the blastema slices can be cultured in basal medium without fetal bovine serum, it was possible to test the response of blastema cells to signaling molecules present in serum, as well as those produced by nerves. We also were able to investigate the response of blastema cells to experimentally regulated changes in BMP signaling. Blastema cells responded to all of these signals by increasing the rate of proliferation and the level of expression of the blastema marker gene, Prrx-1. The organotypic slice culture model provides the opportunity to identify and characterize the spatial and temporal co-regulation of pathways in order to induce and enhance a regenerative response.

## Introduction

Research on regenerating body parts has focused on identifying the signaling pathways involved in initiating and regulating this fascinating and biologically important process. Much of that research has focused on regenerating salamander limbs, and has involved either describing the process of regeneration, or inhibiting regeneration (e.g. by denervating the limb), and then attempting to rescue regeneration. In recent years, the gain-of-function assay for limb regeneration (the Accessory Limb Model, ALM) has identified a number of signals and pathways that are necessary and sufficient for induction of blastema formation and subsequent regeneration of an ectopic limb [1–3]. These experimental approaches have provided insights into the mechanisms of regeneration; however, their utility has been limited by the fact that they all involve regeneration *in vivo*. Thus it is not possible to control the spatial and temporal activation or inhibition of specific pathways without the variability associated with working on a live animal. Attempts to deal with this complexity have involved using *in vitro* culture techniques with either monolayer cultures of dissociated blastema cells, or with blastema explant cultures [4–7]. These studies have been of limited utility because of the limited viability of explants, and the loss of normal blastema cell behaviors (e.g. proliferation) after enzymatic dissociation. To address this challenge, we have adapted organotypic slice culture techniques that have been used widely and successfully in the field of neurobiology [8], and have investigated the response of blastema cells to nerve signals and to BMP2.

Because regeneration is a stepwise process [1,9,10], there are critical signaling events at each step that are required for progression to the next step. Consequently, if the appropriate signaling does not occur at any one of the steps, regeneration will fail to occur [1,9]. Thus each step represents a possible barrier to regeneration, and the failure to overcome at least one of these barriers likely accounts for regenerative failure in mammals [9]. Identifying the steps and discovering how to provide the appropriate signals to progress beyond each step will be required in order to induce regeneration in humans. One or more of the early signals involved in the initiation of regeneration are provided by nerves [1,11–13], which have been the focus of research efforts for decades.

The nerve has long been recognized to be important in regeneration [11–18]. Historically, most experimental work on the role of the nerve has involved *in vivo* studies designed to rescue regeneration of amputated limbs that have been denervated. Limbs that have been denervated fail to initiate regeneration, or fail to progress through the early stages of regeneration [12] to the point where they become independent of the nerve [16]. If the nerves are allowed to regenerate to a point where the supply of nerves exceeds a threshold level, reinjured limbs will be able to regenerate [14,19,20]. Thus attempts to identify the pro-regenerative signals provided by nerves have focused on the later stages of regeneration when the regeneration blastema transitions to becoming independent of nerve signals [21], or on the stage when the regenerating nerve reaches the threshold for the required nerve signaling [7,20,22]. Although denervation experiments have repeatedly demonstrated that a nerve is required (loss-of-function), they have not identified the signals that are required to initiate regeneration and allow for progression through to the late stages at which regeneration is no longer nerve-dependent. The variability associated with *in vivo* experiments (e.g. the age of the animal which affects the timing of the regenerative response, and the degree of nerve regeneration and reinnervation of limbs following denervation) is a challenge to understanding the precise regulation of the nerve signals that are sufficient for inducing regeneration.

To address the challenges associated with studies of regeneration *in vivo*, there have been repeated attempts to isolate and study blastema cells *in vitro*. As with *in vivo* studies, these experiments have provided insights into factors that affect the behavior of blastema cells, particularly with regards to proliferation [4,7,23–25]. Although blastema tissue can be dissociated and blastema cells can be maintained in monolayer culture, the cells quickly lose properties associated with blastema cells *in vivo*. Cultured blastema cells typically have low rate of proliferation and are highly variable; e.g. growth fractions ranging from 0.2–10% [7,26,27] compared to proliferation *in vivo*; e.g. labeling index of 20% to 40% [13,28–30]. The difficulty in maintaining normal behavior of blastema cells *in vitro* has been attributed to their sensitivity to culture conditions such as osmolarity and temperature [5,26,31,32]. Short-term culturing of blastema explants has been reported in studies pertaining to the effect of hormones and growth factors on protein synthesis and cell division [4,23,26,33,34]. The utility of this approach is limited by variability associated with the size of the explants, as well as differences in the viability of cells in the center of the explants compared to cells at the periphery. Finally, dissociated and re-aggregated blastema cells have been cultured in fibrin clots in which they maintain the property of producing high levels of matrix degrading enzymes; however, after a week they lose the ability to signal to other blastema cells in order to induce supernumerary limb patterns when grafted back into a blastema *in vivo* [35].

In order to overcome the challenges of studying the role of the nerve during regeneration *in vivo*, we initiated a systematic assessment of techniques for culturing blastema cells. As reported by others, we observed that the proliferation rates of dissociated blastema cells quickly decreased when the cells were cultured on a range of substrates and culture media, and the cultures could not be maintained and passaged over an extended period of time (data not presented). We therefore reasoned that the behavior of blastema cells *in vivo* was dependent on cell-cell and cell-matrix interactions established during blastema formation. In order to maintain the *in vivo* organization of blastema cells and matrix, we optimized the technique of organotypic slice culture (OSC) that is commonly used in neurobiology. The technique uses a vibrating blade (vibratome) to make serial sections of unfixed blastemas that are of uniform thickness and maintain the original tissue architecture. Multiple sections can be collected from the same blastema, and thus different culture conditions can be tested experimentally without the variability associated with comparing blastema tissues from different animals (e.g. variability in stages of blastemas). We report here that blastema cells in OSC maintain *in vivo* levels of proliferation and tissue architecture, and are responsive to signals from co-cultured dorsal root ganglia explants (DRG), and to exogenous BMP2.

## Materials and Methods

### Ethics statement

This study was carried out in accordance with the recommendations in the Guide for Care and Use of Laboratory Animals of the National Institutes of Health. The experimental work was conducted in accordance with procedures approved by the Institutional Animal Care and Use Committee of the University of California Irvine (IACUC protocol #2007–2705).

### Animal procedures and collection of DRG and blastemas

Axolotls (*Ambystoma mexicanum*) measuring 15–20 cm from snout to tail tip that were spawned at UC Irvine or the Ambystoma Genetic Stock Center at the University of Kentucky were housed on a 12-hour light/dark cycle and fed *ad libitum*. Axolotls were anesthetized using 0.1% ethyl 3-aminobenzoate methanesulfonate salt (MS222, Sigma) at pH 7.0. Limb regeneration leading to formation of medium-late bud stage blastemas was induced by amputation at the level of the mid-humerus.

Details for the culture of dorsal root ganglia (DRG) have been reported previously [36]. Briefly DRG were collected post-euthanasia by surgically removing the spinal nerves that innervated either the forelimb (spinal nerves 3, 4 and 5) or the hind limb (spinal nerves 15, 16 and 17). DRG were cultured individually in 12 well Nunc nunclon plates with 60% L-15, 5% FBS, 1% Insulin, Transferrin, and Selenium (ITS), and gentamicin/amphotericin B (Sigma). Each DRG was attached to the bottom of the culture well by embedding it in a small drop (about 3 μl) of growth factor reduced matrigel (BD Biosciences). Only DRG explants that exhibited neurite outgrowth within the first 24 hours of culture were used for subsequent experiments.

### Organotypic Slice Culture (OSC)

Medium-late bud stage blastemas (mesenchyme and epithelium) were removed surgically from animals, embedded in a 4% agarose gel, and sectioned at a thickness of 250 micrometers using a vibratome. Blastema slices were then cultured on transwell membrane inserts (3.0μm pore) with 6 well plates (BD Falcon) in 60% L-15 media containing 1% Gentamicin, 1% penicillin/streptomycin/amphotericin B, and 1% ITS media supplement (Sigma) in a refrigerated incubator at 19°C. Blastema slices and blastema slice/DRG co-culture were adhered to the transwell membrane with 5μL of growth factor reduced matrigel (BD). Blastema slices receiving additional treatments were cultured in media containing one of the following supplements: 5% Fetal Bovine Serum (FBS, Atlanta Biologicals); 1ng/mL, 10ng/mL, or 100ng/mL recombinant human Bone Morphogenetic Protein 2 (BMP2, Sigma); or 5nM, 50nM, 500nM of the BMP signaling inhibitor, LDN-193189 hydrochloride (Abcam). Half the volume of the culture media was changed every other day. Slices were cultured for 3, 5, or 7 days after being explanted.

### Analysis of cell proliferation and viability

To analyze cell proliferation we used a Click-It EdU labeling kit (Life Technologies) according to the manufacturer’s protocol. The labeling index (% of cells that were in the S phase of the cell cycle) was determined by incubating slice cultures in 80μM of EdU for five hours, which corresponds to approximately 10% of the total cell cycle for blastema cells [37]. The growth fraction (% cells that were proliferating) was determined by incubating the slice cultures in EdU for 80 hours, which corresponds to approximately 1.5 cell cycles (1 cell cycle = 53 hours) [13,37]. After EdU labeling, the slice cultures were washed in PBS, fixed in 4% paraformaldehyde, and sectioned at 5μm. EdU labeled cells were then visualized using the Click-It-EdU alexa-fluor 488 or alexa-fluor 594 kit (Life Technologies), and were counted using the cell counter plug-in for ImageJ. In order to compare the labeling index of blastema cells in response to experimental treatments as compared to the in vivo labeling index, we first injected animals with BrdU two hours before removing the blastema for OCS in vitro. The incorporation of BrdU was visualized by using immunohistochemistry. The total number of cells was visualized by staining with DAPI.

To determine the relative number of viable and dying cells, we used a TUNEL assay kit (Roche) to identify cells that were undergoing cell death. Slides were processed as indicated above and then processed in accordance with the manufacturer’s protocol. For each experiment, the sample size (N) represents the number of biological replicates (independent experiments). For each sample, cells were counted in a total of three sections that spanned the width or thickness of the slice (anterior to posterior or ventral to dorsal). Standard deviation and standard error were calculated using Excel. An F-test was performed to determine equal or unequal variance (i.e. F-value >0.05 or <0.05, respectively). P-values were then calculated using a t-test assuming equal or unequal variance based on the F-value.

### Immunohistochemistry

Blastema slices were fixed for three hours at room temperature in 4% paraformaldehyde, dehydrated in graded alcohol followed by xylene, embedded in paraplast, and sectioned at 5μm. For immunohistochemistry sections were de-paraffinized, rehydrated in TBST, and incubated with anti-acetylated α–tubulin (Abcam, catalog # T7451, diluted 1:250) and anti-RT-97 (DSHB, diluted 1:250) overnight at 4°C. Sections were then washed with TBST, incubated with anti-mouse 594 (Abcam, diluted 1:250) for 4 hours, washed with TBST, and mounted with ProLong Gold Antifade mounting medium with DAPI. Images were obtained using a LSM780 confocal microscope.

The intra-nuclear distribution and translocation of phospho-Smad 1/5/8 was quantified by staining sections with an anti-phospho-Smad 1/5/8 antibody (Millipore, Catalog # AB3848, Lot # 2390361, diluted 1:75). Sections were de-paraffinized and rehydrated in TBST as described above. Heat-induced epitope retrieval was performed at 100°C for 1 hour in Tris/EDTA (pH 9.0). Sections were incubated with anti-phospho-Smad 1/5/8 overnight at 4°C, washed with TBST, and incubated with anti-rabbit 488 (Abcam, diluted 1:250). Twelve regions containing 100–200 mesenchymal cells from three different OSC (N = 3) from each condition tested were selected and analyzed in a double blind manner. The corrected nuclear fluorescence was calculated in order to normalize the intensity of nuclear staining for variation in the area of the nuclei being analyzed as well as for the background fluorescence [38].

### Quantitative PCR

Blastema slice RNA was extracted using Trizol (Invitrogen) in conjunction with the Nucleospin RNA XS kit (Macherey-Nagel). Samples were processed to generate cDNA using the Transcriptor First Strand cDNA Kit (Roche). Real-time PCR was performed using a LightCycler 480 (Roche) and the resulting data were analyzed using the Pfaffl method [39]. Sequences for the primers for qPCR (Sigma) were based on primers published previously [40,41].

GAPDH FWD: GACGCTGGTGCAGGCATTGCC

GAPDH REV: ACCATCAGGTCCACAACACGCTGAC

Prrx1 FWD: GGCGAAAGTTTGCTCTTCGG

Prrx1 REV: GGCGAAACTTTGCTCTTCGG

## Results

### Organotypic slice cultures of axolotl blastemas remain viable for several days

**Fig 1:**
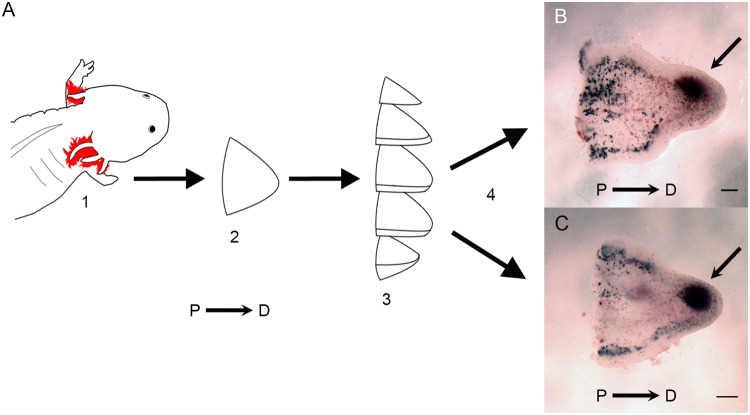
Organotypic slice culture model for axolotl blastemas. (A1) The limb of an axolotl (*Ambystoma mexicanum*) was amputated and allowed to regenerate a medium-late bud stage. (A2) The blastema was surgically removed from the animal, (A3) was sectioned using a vibratome (A3) and cultured (A4, B, C). Depending on the size of the blastema, a typical blastema yielded 3–4 slices that appeared similar when cultured (B and C). A region of pooled blood cells in the apical region of adjacent slices is indicated by the arrows in (B and C). The proximal (P) to distal (D) orientation of the sections is indicated. Scale bars = 500μm.

OSC allowed for the culture of multiple sections of uniform dimensions from a single blastema (Fig 1). In response to limb amputation, a blastema (medium-late bud stage) formed in about 10–12 days (Step 1, Fig 1A). The blastema was surgically removed (Step 2), embedded in gelatin and sectioned using a vibratome that resulted in uniform 250μm thick longitudinal sections (Step 3). Depending on the size of the blastema, a typical mid bud blastema from an adult axolotl (10-15cm) yielded 3–4 slices that were similar in shape and size. Adjacent blastema slices maintained the tissue architecture of *in vivo* blastemas (i.e. mesenchyme surrounded by a wound epithelium) (Fig 1B and 1C). The blastema slices were then cultured under various experimental and control conditions (Step 4).

**Fig 2:**
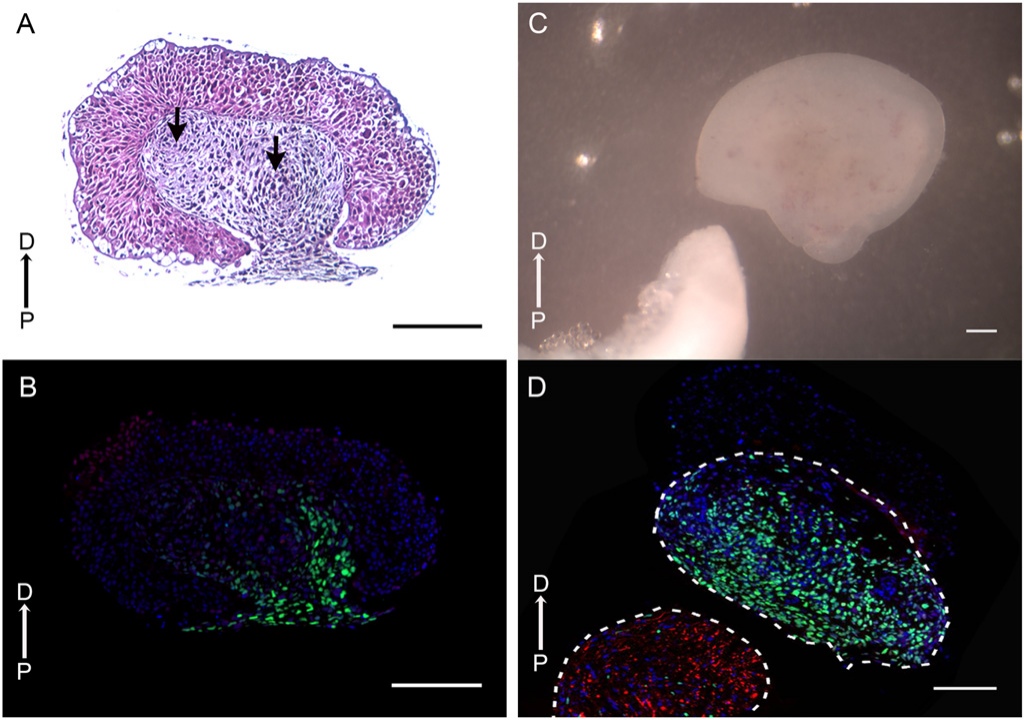
Organotypic blastema slices survive and proliferate *in vitro*. (A) Hematoxylin and eosin stained section of a blastema slice that had been cultured for 10 days in the presence of 5% FBS. Regions of pre-cartilage condensations are evident (arrows). The blastema epithelium has begun to migrate over the proximal cut end. (B) EdU labeling of a blastema slice after 10 days *in vitro*. EdU positive nuclei (green) are present in the mesenchyme but absent from the epithelium. (C) A bright field image of a blastema slice (upper right) and DRG (lower left) after 10 days of co-culture. (D) Immunofluorescence of the blastema slice/DRG co-culture illustrated in (C). Proliferating cells in the slices (green) are restricted to the mesenchyme, nuclei are stained with DAPI (blue), and neurofilaments are labeled with RT97 (red). The proximal (P) to distal (D) orientation of the sections is indicated. Scale bars = 500μm.

Blastema slices appeared healthy and the cells continued to proliferate *in vitro* over an extended period of time (Fig 2). After 10 days in culture, the slices maintained a normal morphology with a thickened epithelium surrounding the blastema mesenchyme (Fig 2A). The cells within the slice appeared healthy and regions of high cell density corresponding to pre-chondrogenic condensations were evident in some sections (Fig 2A, arrows). Although the appearance of an OSC blastema was similar to a blastema that was integrated into the amputated limb (e.g. there was an apical epithelium that covered the blastema mesenchyme), there also were differences. At the proximal boundary of the slice, the epidermis began to migrate over the free surface that was created when the blastema was surgically removed from the animal. This phenomenon referred to as “epiboly”, has been described previously in whole-mount explants of amphibian blastemas and limb buds [42,43]. Many of the cells of the blastema mesenchyme incorporated EdU after 10 days of culture, although cells in the regions of high cell density (visualized by DAPI staining of the nuclei) did not (Fig 2B and 2D). The keratinocytes of the apical epithelium (Apical Epithelial Cap, AEC) in this experiment (cultured with 5% FBS) did not incorporate EdU (Fig 2B), which was consistent with the observation that AEC cells withdraw from the cell cycle *in vivo* [44,45]. Better understanding how the proliferative response of AEC keratinocytes, particularly the basal keratinocytes [2] is regulated in organotypic slice cultures is a goal of future experiments.

As reported previously, DRG can be isolated and co-cultured with blastema explants in order to study the interaction between nerves and blastema cells [36]. To investigate the response of OSC blastema cells to signaling from nerves, blastema slices were co-cultured with DRG (Fig 2C and 2D). Both OSC blastema slices and DRG explants appeared healthy after an extended period of time (10 day cultures illustrated in Fig 2C and 2D). As with blastema cultures without DRG explants, many of the mesenchymal cells not associated with regions of high cell density incorporated EdU (Fig 2D, green). As with blastema slices cultured without DRG, the keratinocytes of the AEC did not incorporate EdU. As reported previously [36], the cultured DRG contained large numbers of regenerating neurons with RT97-positive neurofilaments (Fig 2D, red).

**Fig 3:**
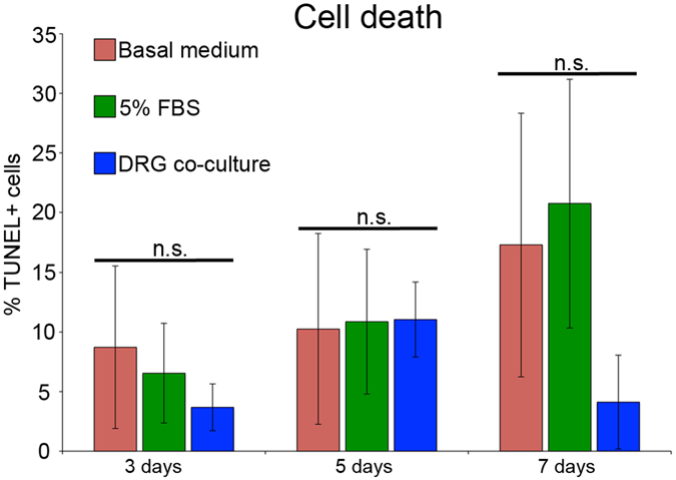
Cell death in blastema slices after culture for 3, 5, and 7 days. The percentage of TUNEL positive cells was not significantly different between treatments at any of the time points, or between the three time points. Error bars represent one Standard Deviation of the Mean, and P-values were determined by t-test with 2 tails assuming unequal variances. The number of biological replicates for 3, 5, and 7 days were as follows: Basal medium: 5, 5, and 3, 5%FBS: 4, 4, and 5, DRG co-culture: 3, 3, and 3.

As noted above, blastema slice cultures appeared healthy for at least 10 days in culture. During this period, some pyknotic nuclei were observed in cells along the proximal edge of the explant that was created when the blastema was surgically removed from the animal. TUNEL staining was relatively low, and was not significantly different at a given time point for slices cultured with or without FBS or co-cultured DRG (Fig 3). As with the distribution of pyknotic nuclei, most of the TUNEL-positive nuclei were restricted to the region immediately adjacent to the proximal boundary of the explant. After five days *in vitro*, TUNEL staining remained about the same as observed at three days, and did not differ depending on the culture conditions. After seven days *in vitro*, TUNEL staining was more variable and the percentage of TUNEL-positive nuclei was higher in slices cultured in basal medium and 5%FBS; however, this increase was not significant. Based on these data, we did not culture slices for longer than seven days *in vitro* so as to minimize variability associated with cell death. This time period is comparable to the window for experimental work using OSC in neurobiology [8].

**Fig 4:**
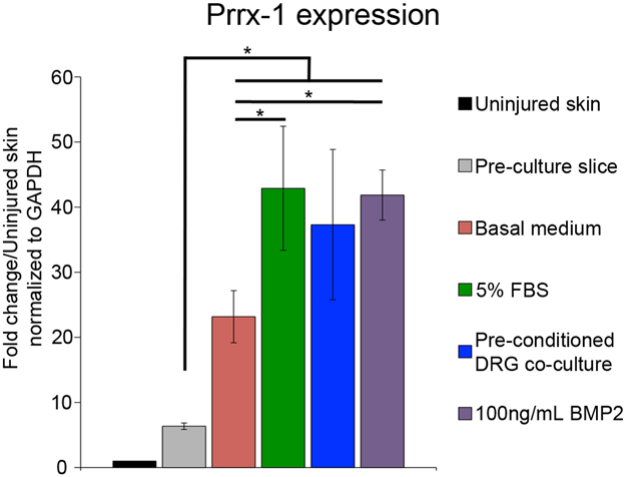
Prrx-1 expression in blastema slices. Fold change in Prrx-1 levels after seven days of culture under different culture conditions. The value for “Uninjured Skin” was determined for samples of skin that had not been cultured. The value for “Pre-culture Slice” was determined for blastema slices that had not been cultured. Error bars represent S.E.M., and P-values were determined by t-test with 2 tails assuming unequal variances. The number of biological replicates for the conditions tested were as follows: Pre-culture slice: N = 5, Basal medium: N = 7, 5%FBS: N = 5, Pre-conditioned DRG: N = 5, 100ng/mL BMP: N = 3. Each biological replicate consisted of four technical replicates. Asterisk (*) = P<0.05.

Expression of Prrx-1 (Paired-related Homeobox 1) in OSC blastema mesenchymal cells was maintained and increased *in vitro* (Fig 4). Prrx-1 is a transcription factor that is expressed at high levels in developing and regenerating axolotl limb mesenchymal cells [41], but is expressed at low levels that are not detected by *in situ* hybridization in uninjured skin. Expression of Prrx-1 in the blastema is regulated by interactions between the wound epithelium and the nerve [41], and thus it is a marker for regenerating blastema cells [3,41,46]. As reported previously [47], Prrx-1 expression was detected at low levels by qPCR in uninjured skin (Fig 4). Expression in blastema sections prior to being culture was significantly upregulated an average of 6-fold, which is comparable to the increased level of expression *in vivo* when comparing blastemas to uninjured skin [3]. Blastema slices cultured in basal medium for 7 days expressed Prrx-1 at a 23-fold higher level relative to uninjured skin (about a 4-fold increase during the culture period. Since Prrx-1 expression is restricted to the distal tip of blastemas *in vivo*, this increase in expression *in vitro* could be a result of increased numbers of cells in the slices being induced to express this gene rather than an increase in the level of expression in the distal blastema cells. Better understanding the mechanisms of Prrx-1 expression during regeneration is a goal of future experiments using OSC. Taken together, these data indicate that blastema slices cultured in basal media are viable and maintain expression of a gene that is characteristic of regenerating blastema cells.

### Organotypic slice cultured blastema cells responded to FBS and co-culture with DRG

Because blastema cells remained viable when cultured in a minimal basal medium, it was possible to assay for their response to changes in the culture environment. As reported above, there was no significant change in the percentage of TUNEL-positive cells in basal medium when compared to cultures with added FBS or with co-cultured DRG. Expression of Prrx-1 was maintained, and increased over time, in slices cultured in basal medium (Fig 4). Addition of 5% FBS to the basal medium induced a significant increase in the level of Prrx-1 expression after seven days in culture (43-fold relative to uninjured skin; 7-fold relative to an uncultured blastema; nearly 2-fold relative to blastema slices cultured in basal medium). While there was an increase in Prrx-1 expression in slices co-cultured with a DRG compared to slices cultured in a basal medium (1.6-fold), the difference was not statistically significant.

**Fig 5:**
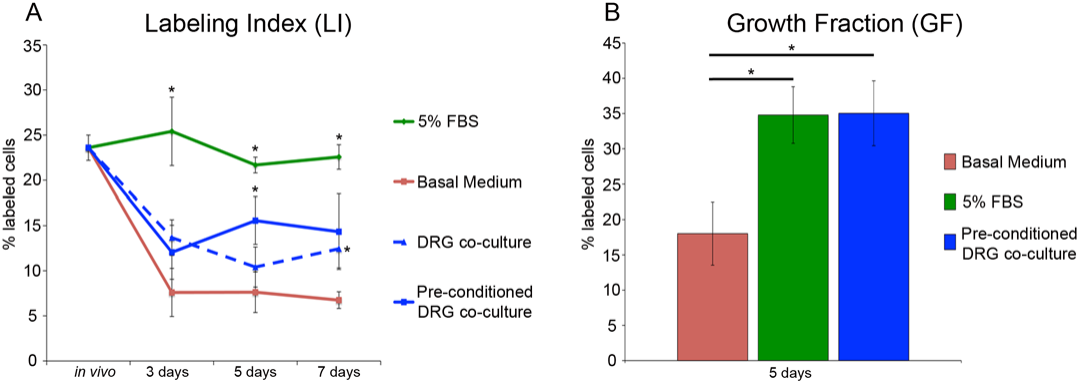
Labeling index and growth fraction in blastema slices. A) Labeling index of blastema mesenchymal cells in blastema slices over a period of seven days in culture under different culture conditions as indicated. Labeling period was for five hours. The value for “*in vivo*” was determined by injecting animals with BrdU two hours prior to collecting the blastema for sectioning and counting of labeled cells without culture. Error bars represent S.E.M., and P-values were determined by T-test with 2 tails assuming unequal variances. The number of biological replicates for *in vivo*: N = 22. The number of biological replicates for the following conditions on 3,5, and 7 days were as follows: Basal medium: N = 6, 8, and 9. 5%FBS: N = 7, 7, and 7. DRG co-culture: N = 3, 5, and 6. Pre-conditioned DRG: N = 5, 5, and 4. Asterisk (*) = P<0.05 when compared to the LI in basal medium at equivalent time point. B) Growth Fraction of blastema mesenchymal cells in blastema slices after five days of culture under different culture conditions as indicated. Labeling period was for 80 hours. Error bars represent S.E.M., and P-values were determined by t-test with 2 tails assuming unequal variances. N = 4. Asterisk (*) = P<0.04.

In contrast to Prrx-1 expression that increased during culture in basal medium, proliferation of the blastema mesenchymal cells in basal medium decreased during the initial three days in culture and remained at a relatively low level over the next four days (Fig 5A). The labeling index (LI) of the mesenchymal cells in blastema slices prior to amputation (equivalent to *in vivo*) was about 23%, which was comparable to values reported previously for blastema cells *in vivo* [13,28–30]. In basal medium the LI had decreased to about 7% after three days *in vitro*, and remained constant at this level until the end of the experiment (seven days). This decrease in proliferation was comparable to the rate of proliferation observed *in vivo* in denervated newt limbs [48] and regressing axolotl ectopic blastemas [1].

The rate of proliferation was maintained at *in vivo* levels in cultures that were supplemented with 5% FBS (Fig 5A). The proliferation rate in slices that were co-cultured with a DRG removed from animals the same day as initial culturing of OSC was higher (not statistically significant) than the rate for slices cultured in basal medium during the first five days of culture. After seven days of culture, the LI in blastema/DRG co-cultures was about 2-fold higher compared to the basal medium cultures (p = 0.015).

Gene expression data suggest that DRG might undergo a refractory period or injury response that changes the expression of genes involved with growth and proliferation of blastema cells [36]. It also has been shown that the growth response to DRG was greatest when DRG were surgically grafted 2–3 days prior to whole explant culture of blastemas rather than concurrently, supporting the idea that DRG might require a period of recovery before the growth promoting effect of the nerve is restored [4]. In light of these observations, we hypothesized that DRG might require a recovery period prior to co-culture with DRG in order to restore the growth promoting effect of the nerve. Because previous work has shown that there is a large change in gene expression when comparing DRG *in vitro* for 5 days to day 0 DRG [36], we decided to pre-condition DRG for 5 days prior to co-culture with OSC. Pre-conditioning DRG led to a higher proliferation rate in OSC at days 3, 5, and 7 when compared to OSC cultured in basal medium (Fig 5A). While the proliferation rate was higher at all three time points, it was at its highest and most significant at day five, when the LI was approximately 15% (Fig 5A).

The decrease in LI in basal medium cultures likely was not a consequence of cell death since the percentage of TUNEL-positive nuclei was not significantly different between the different culture conditions or between the various time points of the experiment (Fig 3). However, it appeared that a significant number of cells withdrew from the cell cycle when cultured in basal medium (Fig 5B), which could account in part for the decrease in the LI. The growth fraction (80 hours of continuous EdU labeling which corresponds to 1.5 cell cycles) of blastema cells cultured in basal medium was significantly lower (about 50% decrease) that that of cells cultured in medium with 5% FBS or co-cultured with pre-conditioned DRG in basal medium (both about 35%) (Fig 5B). Although the growth fraction (about 18%) decreased in the basal medium cultures without FBS or co-cultured with DRG, it was considerably higher and more consistent that what has been reported for monolayer cultures of blastema cells (growth fraction of 0.1%- 9% after 96 hours of labeling [7].

### Organotypic slice cultured blastema cells respond to human BMP2

A major advantage of OSC is that the cells remain viable when cultured in minimal basal medium, and it therefore is possible to test the affects of activating specific signaling pathways. Our previous analyses of the transcriptional response of cultured DRG identified BMP2 as a signaling molecule whose expression was specifically upregulated in response to co-culture with a blastema explant [36]. We therefore hypothesized that a regenerating nerve produces BMP2 in response to interacting with blastema mesenchymal cells, and that BMP2 in turns regulates the behavior of the blastema cells.

**Fig 6:**
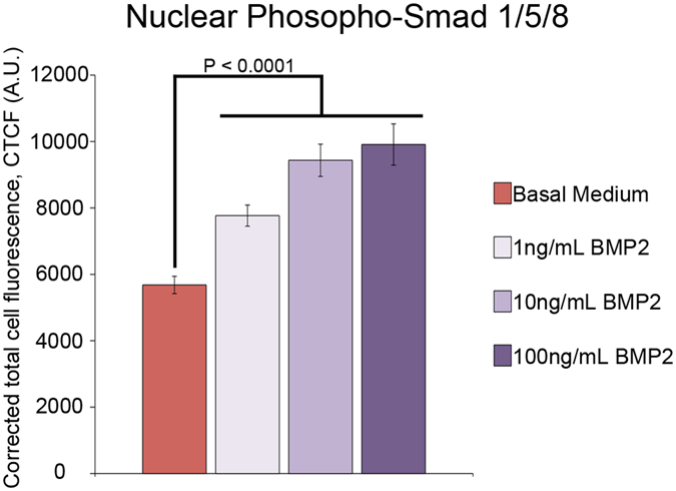
Nuclear P-Smad 1/5/8 fluorescence in response to BMP2. Nuclear Phospho-Smad 1/5/8 staining in blastema mesenchymal cells in blastema slices cultured in the presence or absence of exogenous human BMP2 in amounts as indicated. The corrected nuclear fluorescence was calculated in order to normalize the intensity of nuclear staining for variation in the area of the nuclei being analyzed as well as for the background fluorescence [40]. Error bars represent S.E.M., and P-values were determined by t-test with 2 tails assuming unequal variances.

To test whether cultured blastemas cell respond to exogenous BMP2, we added recombinant human BMP2 to the basal medium of OSC blastema cells at concentrations that span our best estimate for the *in vivo* concentration of BMP2 [49–53]. We first determined that the axolotl blastema cells responded to the addition of non-axolotl BMP2 by quantifying the changes in the level of phospho-Smad 1/5/8 (p-Smad 1/5/8) immunofluorescence within the nucleus of OSC blastema cells (Fig 6). Treatment with BMP2 at all three concentrations tested (1–100 ng/ml) induced a significant increase (nearly double at 100 ng/ml) in the amount of nuclear-localized p-Smad 1/5/8 relative to basal medium, indicating that human BMP2 activated the canonical BMP signaling pathway in axolotl blastema cells.

**Fig 7:**
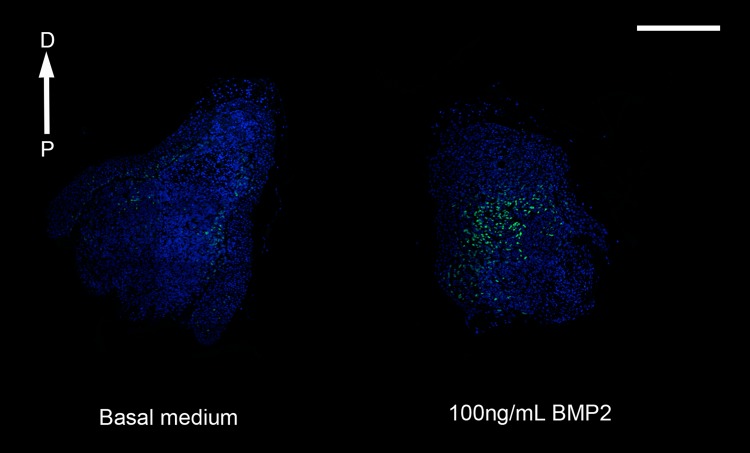
Proliferation in response to BMP2. Immunofluorescence showing EdU labeling of blastema slices originating from the same blastema and cultured in either basal medium or 100ng/mL BMP2. EdU positive proliferating cells are green, nuclei are stained with DAPI and are blue. The proximal (P) to distal (D) orientation of the sections is indicated. Scale bar = 1mm.

**Fig 8:**
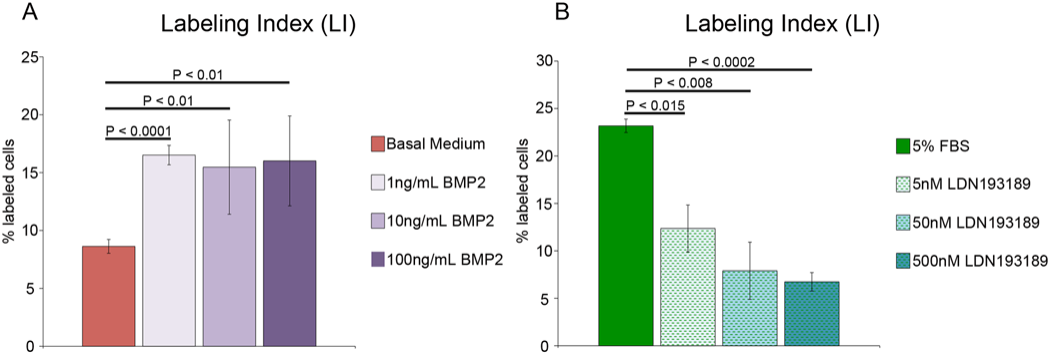
Labeling index in response to exogenous BMP2 and LDN193189. A) Labeling index of blastema mesenchymal cells in blastema slices over a period of five days in culture in the presence or absence of exogenous human BMP2 in amounts as indicated. The number of biological replicates = 4. B) Labeling index of blastema mesenchymal cells in blastema slices over a period of 5 days cultured in 5%FBS and in the presence or absence of LDN193189 in amounts as indicated. The number of biological replicates = 3. Error bars in both A and B represent S.E.M. and P-values were determined by t-test with 2 tails assuming unequal variances.

OSC blastema mesenchymal cells responded to increased BMP2 signaling by increasing expression of Prrx-1 to the level observed in response to 5% FBS (Fig 4). In addition, the rate of proliferation increased significantly (double that for cultures in basal medium) at all three concentrations of BMP2 that were tested (Figs 7 and 8A). The average labeling indices for all three concentrations were comparable to each other and to *in vivo* rates of proliferation [13,28–30]. Since the mean LI did not change in response to increasing dose, and the variability (standard error) was considerable smaller at the lowest dose tested, we concluded that 1 ng/ml BMP2 was an appropriate dose for experiments with OSC axolotl blastema cells.

To determine if the BMP2 pathway was indeed involved with proliferation in OSC, we used the BMP2 pathway inhibitor LDN193189 in order to abrogate the effect that 5%FBS has on the proliferation rates of OSC given that BMP is present in FBS [54]. LDN193189 is a small molecule derivative of Dorsomophin that prevents the BMP induced Smad1/5/8 phosphorylation by binding to the ATP binding site in the kinase domain of Type I BMP receptors [55–57]. We found that incubation of OSC in medium containing 5% FBS along with LDN193189 at doses of 5, 50 and 500nM all had a significant effect on the rate of proliferation in slices (Fig 8B). At LDN193189 doses of 50nM and 500nM, the LI was reduced to the level observed in OSC cultured in basal medium (Fig 8B).

## Discussion

### Maintenance of in vivo blastema cell behavior is dependent on maintenance of cell-cell and cell-matrix interactions

Amphibian cells, including those derived from salamanders, have been used for *in vitro* experimentation since the advent of modern cell culture [58]. Early experiments using salamander tissues typically employed the technique of whole explant cultures, either using the developing limb bud or regenerating limb blastema [4,24,32,43,59–61]. While these experiments paved the way for the study of regeneration *in vitro*, the use of explant cultures has its drawbacks; namely, increased cell death, lack of reproducibility owing to the variability of sample source and preparation, and lack of access to the interior mesenchymal tissues for experimental manipulation when blastemas are cultured with the epithelium present [62]. In addition, normal rates of proliferation are not maintained. For example, medium bud blastemas from newts were reported to have a mitotic index of 1.2% (number of cells in the M phase of the cell cycle relative to the total number of cells) that decreased to 0.2% when the blastemas were placed in explant culture [24].

The technique of dissociation, culture and passage of individual cells is an alternative approach to *in vitro* studies that avoids the drawbacks of explant cultures. Although blastemas can be dissociated and blastema cells can be cultured individually, the cells quickly lose the behavior of blastema cells *in vivo*, most notably they have a dramatically lower rate of proliferation. Data for the labeling index (number of cells in the S phase of the cell cycle relative to the total number of cells) of blastema cells *in vitro* are comparable to uninjured skin (2%) in contrast to that of blastema cells *in vivo* (20%- 40%) [7,13,26–30]. It appears that this decrease in proliferation is in part a consequence of cultured blastema cells exiting the cell cycle when cultured. For example, the percentage of blastema cells that incorporated BrdU after 96 hours of continuous labeling (growth fraction) was highly variable (0.1% to 9%, with a median of 1%) [7] in contrast to a growth fraction of 40% to 80% (3H-thymidine injection every 12 hours for two to four days) for blastema cells *in vivo* [48]. With OSC (this study), the growth fraction (80 hours labeling which corresponds to approximately 1.5 cell cycles) was 35% after five days in culture (Fig 5B) and as high as 63% after 10 days in culture (N = 3 samples). Consistent with maintaining a growth fraction comparable to blastema cells *in vivo*, the labeling index of OSC blastema cells cultured with FBS also was comparable to what has been reported *in vivo* (Fig 5A).

We hypothesize that normal blastema cell behavior is dependent on maintaining normal cell-cell and cell-matrix interactions. OSC allows for cell-cell and cell-matrix interactions to occur, as well as providing conditions for all of the cells to be uniformly exposed to the cell culture media and experimental supplements [8,62]. Consistent with this hypothesis are the findings that blastema cells remain viable with only a low amount of cell death, that they continue to express a blastema marker (Prrx-1), that they continue to proliferate at *in vivo* levels, that their rate of proliferation is responsive to the presence of nerve signals provided by DRG, and that they can respond to changes in the culture environment (addition of FBS or BMP2).

There are several likely reasons why OSC is an appropriate model for studying the biology of blastema cells. First, a regenerating limb blastema is comprised of a heterogeneous population of progenitor cells [63,64], which has been noted previously as to why culture techniques requiring dissociation are unadvisable [32]. In addition, blastemas are not only heterogeneous in terms of cell types of different lineages, but also in terms of the state of developmental plasticity of blastema cells. The cells in the apical region of a medium-late bud blastema are undifferentiated and developmentally plastic, in contrast to those at more basal regions that are beginning to redifferentiate and have stabilized their positional information [65,66]. The interaction of blastema cells with different positional information controls growth and pattern formation during regeneration [1,67,68], and thus the spatial relationship of blastema cells would be expected to be important in terms of regulating their behaviors. Dissociation of a blastema into single cells disrupts the spatial organization of the blastema; whereas, OSC maintains the normal positional interactions. The stability of positional information is maintained for about a week in posterior blastema cells cultured as a micromass in a fibrin clot and grafted into the anterior of a host blastema *in vivo* [35]. Testing the hypothesis that the spatial organization of positional information also is maintained in OSC is a goal of future experiments.

### Blastema cell gene expression and proliferation are responsive to nerve signals and to BMP2

A distinct advantage to organotypic slice cultures is that cultured blastema cells remain viable without the addition of FBS. Amphibian tissues are well suited for organ culture [69,70], and early experiments utilizing salamander tissues reported proliferation and the long-term survival of cells in basic salt solutions [32,43,59]. OSC blastema cells also survived and proliferated when cultured in basal medium with FBS. The mitogenic activity of FBS led to its use in early studies to identify mitogenic factors, and subsequently to general use in cell culture in order to expand cell populations for passage. It has been noted that FBS evokes a specific physiological response in cultured cells that is reminiscent of wound healing [71]. Cells such as fibroblasts, which are the progenitors of many of the cells found in the blastema [63,64], would encounter the factors found in FBS in the early wound environment created by tissue injury. Thus OSC blastema cells respond to FBS and proliferate at *in vivo* levels (Fig 5A), even when a nerve is not present. In spite of the response of OSC cultured blastema cells to added FBS, the utility of OSC for studying the behavior of blastema cells is that addition of FBS is not necessary; therefore, it is possible to study the mechanisms by which specific signaling pathways are involved in the regenerative response.

One pathway that likely is involved in the regulation of blastema cell behavior is BMP signaling, which previously has been implicated in controlling regeneration in a number of model systems, including mammals [36,47,72–74]. The OSC model allowed us to determine that axolotl blastema cells responded to a specific, optimal dose of human BMP2 (1 ng/ml) by increasing the expression of a blastema marker gene (Prrx-1) and increasing the rate of proliferation. Furthermore, incubation with the BMP pathway inhibitor, LDN193189, decreased the rate of proliferation similar to one seen in OSC cultured in basal medium. In addition, we have identified a similar response (changes in gene expression and proliferation) to signaling from co-cultured nerves, which has been associated previously with FGF as well as BMP signaling [21,36,47]. The report that an ectopic blastema can be induced by implanting beads soaked in a cocktail of FGF2, FGF8 and GDF5/BMP2/BMP7 in the absence of a deviated nerve provides additional evidence that blastema formation can be induced by the activation of well characterized and highly conserved signaling pathways (e.g. FGF and BMP) [47]. The OSC model provides the opportunity to discover the mechanism for the spatial and temporal co-regulation of these pathways in order to induce and enhance a regenerative response.
